# Saving faces is not enough: Mental health must become core noma care

**DOI:** 10.1371/journal.pmen.0000723

**Published:** 2026-09-10

**Authors:** Anil Fastenau

**Affiliations:** 1 Department of Global Health, Institute of Public Health and Nursing Research, University of Bremen, Bremen, Germany; 2 Marie Adelaide Leprosy Centre (MALC), Karachi, Pakistan; 3 German Leprosy and Tuberculosis Relief Association (GLRA/DAHW), Wuerzburg, Germany; 4 RedAid Nigeria, Enugu, Nigeria; PLOS: Public Library of Science, UNITED KINGDOM OF GREAT BRITAIN AND NORTHERN IRELAND

## The second neglect of noma

Noma has traditionally been described through some of the most disturbing outcomes in global health: rapidly progressing destruction of the mouth and face, severe functional impairment, and death [1]. In 2023, the World Health Organization (WHO) formally recognized noma as a neglected tropical disease (NTD), an overdue acknowledgement of a disease concentrated among some of the world's poorest and most marginalized children [1]. Yet another dimension of noma remains neglected: the mental health of those who survive it.

For decades, success in noma care has understandably centered on survival, wound healing and reconstructive surgery. But survival is not recovery. A child may survive noma and carry its consequences for decades: altered facial appearance, difficulties eating or speaking, repeated operations, exclusion from school, ridicule, discrimination, restricted opportunities for employment and relationships, and internalized stigma [2–4]. If health systems reconstruct faces while leaving these consequences untreated, we have addressed only part of the disease.

The emerging evidence should make this position increasingly difficult to defend. A 2026 study of 244 children aged 6–16 years with noma-related facial disfigurement attending the Noma Children's Hospital in Sokoto, Nigeria, found clinically significant depressive symptoms in 76.6% of participants [5]. Girls were particularly affected [5]. Although these findings derive from a single specialist-center population with severe sequelae and screening cannot be equated with psychiatric diagnosis, the magnitude of psychological distress demands attention rather than further evidence before action. Research from Ethiopia similarly documents substantial psychosocial and functional morbidity among people living with untreated noma sequelae [6].

Noma therefore produces what might be called a second neglect. The first neglect allows preventable and treatable disease to progress because poverty, malnutrition, weak health systems and delayed access to care remain unaddressed. The second begins after survival, when visible disfigurement, stigma, psychological distress and social exclusion are treated as secondary consequences rather than integral components of the disease zburden.

This distinction matters because mental health should not become another vertical noma programme.

## Integrating mental health into noma care

The countries where noma occurs often have severe shortages of specialized mental-health professionals. Creating stand-alone psychiatric services for a comparatively rare and geographically dispersed disease would consequently be neither realistic nor sustainable. Fortunately, it is also unnecessary.

WHO's 2026 Essential care package to address mental health and stigma for persons affected by neglected tropical diseases provides a different direction: integrate mental-health care, psychosocial support and stigma reduction into NTD programs, community services and primary care [7]. This approach is particularly relevant for diseases associated with visible differences and disability, including noma, leprosy, cutaneous leishmaniasis, Buruli ulcer and lymphatic filariasis. WHO's integrated skin-NTD framework similarly provides an existing platform for combining case detection, disability prevention, stigma reduction, rehabilitation and person-centered care [8].

Noma should enter these systems rather than sit beside them.

In endemic settings, frontline workers trained to recognize noma could also be trained to recognize psychological distress. Primary-health-care services could incorporate brief validated mental-health screening into assessment and follow-up. Clear referral pathways could connect people requiring more intensive care with available mental-health services. Existing leprosy and skin-NTD programs could expand peer-support groups, counselling, self-care groups and stigma-reduction interventions [9,10] to include people affected by noma. Community health workers could support families as well as patients, because stigma, caregiving burdens and poverty rarely affect the individual alone [11].

## Psychosocial support across the continuum of care

Psychosocial care should furthermore begin before reconstructive surgery, continue during treatment and extend afterwards. Surgery can restore function and appearance, but it cannot automatically reverse years of rejection, interrupted schooling, social withdrawal or internalized shame. Mental-health outcomes, social participation and quality of life should therefore become legitimate indicators of successful noma rehabilitation alongside surgical and clinical outcomes.

## Meaningful involvement of people with lived experience

Integration must also mean moving beyond seeing people affected by noma solely as recipients of services. Survivors should participate in designing programs, training health workers, community education, stigma reduction, peer support and research [12]. Experiences from Nigeria and Burkina Faso illustrate how noma prevention, early detection and care can build on existing primary-health-care and community platforms rather than creating parallel disease-specific systems [13,14].

This is not merely an ethical argument. People with lived experience understand dimensions of stigma, concealment, health-seeking and social reintegration that health professionals may never see. Their participation can make services more acceptable and expose outcomes that conventional clinical indicators miss.

## Redefining successful noma care

Research priorities must change accordingly. We need longitudinal studies assessing depression, anxiety, stigma, social participation, education, quality of life and family wellbeing before and after medical and surgical interventions. Yet the absence of perfect evidence must not become an excuse for inaction. The available evidence already establishes that psychosocial morbidity is substantial, while WHO now provides an implementation framework for integrated NTD mental-health care [5–7].

The recognition of noma as an NTD created an opportunity to redefine what successful noma control means. Preventing disease and ensuring early treatment remain paramount. Reconstructive surgery will remain indispensable for many survivors. But the endpoint cannot simply be a closed wound or reconstructed face. A health system should not claim to have treated noma until it has also addressed the person's opportunity to live, learn, participate and belong. Mental health and psychosocial support are not optional additions to noma care. They are noma care.
